# The impact of Croatia’s 2013 Primary Care Payment Reform: an exploratory case study of a rural family practitioner group practice

**DOI:** 10.3325/cmj.2025.66.360

**Published:** 2025-10

**Authors:** Tatjana Prenđa Trupec, Milena Pavlova, Tetiana Chernysh, Wim Groot

**Affiliations:** 1 Care and Public Health Research Institute, Maastricht University, Maastricht, the Netherlands; 2 Maastricht University, Maastricht, the Netherlands

## Abstract

**Aim:**

To explore the effect of Croatia’s 2013 Primary Care Payment Reform on patient care.

**Methods:**

The performance of one rural family practitioner group practice in Breznica was assessed one year before and one year after implementation. Using an exploratory case study design with quantitative methods, we examined outcome variables linked to the new payment model. Drawing on publicly available activity reports, we conducted linear regression analyses to identify potential associations.

**Results:**

We observed changes in most incentivized outcomes, notably increases in preventive check-ups and decreases in hospital referrals. Some indicators, such as the increase in prescribing despite financial disincentives, remain inconclusive and highlight the need for further investigation.

**Conclusions:**

These findings are preliminary but provide valuable hypotheses for guiding more rigorous, longitudinal, and multi-site studies.

Over the past several decades, numerous initiatives aimed at strengthening primary health care have been implemented across European countries (1). Many of these initiatives involved changes to financial incentive schemes or the introduction of quality and performance indicators, linking family practitioners’ rewards to the achievement of specific targets (2,3).

Evaluations of these reforms are still inconclusive. One study found that “the introduction of financial incentives was associated with substantial apparent quality improvement for incentivized conditions” (4). A survey of pay-for-performance (P4P) models in the health sector across 11 countries highlighted significant variation in how extensively financial incentives are used to improve the quality of care in primary health care settings (5). Nevertheless, gaps remain in the literature regarding the challenges and enabling factors involved in implementing complex interventions within primary care (6). Overall, the current evidence base is insufficient to draw firm conclusions about the effectiveness of financial incentives in enhancing the quality of primary health care (7).

Since the early 1990s, Croatia has implemented several reforms, with capitation serving as the central payment mechanism (8). Prior studies have highlighted challenges such as out-of-pocket payments and pressures on system sustainability (9,10). The 2008-2010 payment reforms aimed to contain costs and improve efficiency by introducing a new payment model for family practitioners, in which 80% of income depended on capitation and the remaining 20% on activity-based measures (11). However, the large proportion of income tied to capitation continued to negatively affect referrals to hospitals. Over the years, Croatia has experienced a persistent shortage of family practitioners, who are also unevenly distributed across the country. Many rural areas, including 60 inhabited islands, have faced significant challenges in accessing health care, which results in varying quality of care provided (12). To tackle these issues, the Croatian Health Insurance Fund, the single public payer of health services, introduced a payment reform for primary health care in 2013. The reform included switching from payment almost mainly based on capitation to mixed payment based on capitation, P4P with financial incentives for key performance and quality indicators, fee for service, and fixed costs. The effects of this reform have not been systematically assessed. The present study addresses this gap by conducting an exploratory case study of Croatia’s 2013 primary health care payment reform, with the aim of examining its outcomes. We conducted an exploratory case study of a rural group practice in Breznica, Croatia, which was actively involved in the design of health care reforms. The aim is to generate hypotheses regarding how financial incentives may influence physicians’ behavior and patient care within this group practice.

## Methods

### Design and period

We used an exploratory single-case, longitudinal pre-post design to compare changes in outcomes of the study practice between the 12 months before the primary health care financing reform (April 1, 2012-March 31, 2013) and the 12 months after its introduction (April 1, 2013-March 31, 2014). Since the aim of this study was to examine differences in health care delivery within a single group practice before and after the reform, data on family practitioners’ activities were extracted from publicly available reports of the Breznica group practice. These activities included drug prescribing, hospital referrals, preventive check-ups, the incidence of newly diagnosed non-communicable diseases (NCDs), and other relevant indicators.

### Study population

Patient-level data for this exploratory case study were obtained from publicly available activity reports of a private rural group practice in northern Croatia, covering Breznica and the surrounding area (approximately 3500 inhabitants). The practice, contracted by the Croatian Health Insurance Fund, was an early participant in the 2013 financing reform. The clinical team consisted of two family physicians (one male, 53 years old, and one female, 47 years old at the beginning of the analyzed period), supported by nursing and administrative staff, serving 3036 registered patients of all ages. Anonymized data were provided by the family practitioners upon request, and the family practitioners were informed of the study’s aims. Analyses included all patients who visited the practice at least once between 2012 and 2014.

### Study setting

By 2013, the financing of family medicine in Croatia was structured around three main components: approximately 90% of physicians’ income derived from capitation payments, about 9% from fee-for-service reimbursement covering roughly ten defined services, and the remaining 1% from administrative payments, primarily intended to support informatization. In the same year, a payment reform was introduced, establishing a new financing model composed of five core elements that combined fixed and variable components. The fixed component covered physician and nurse salaries, practice-operating costs, and age-adjusted capitation, with additional weighting applied for specialists in family medicine.

The variable component included approximately 200 diagnostic-therapeutic procedures (DTPs), both preventive and curative, subject to an upper revenue limit. In addition, an incentive component was introduced, based on key performance indicators (KPIs) and quality indicators (QIs), each accounting for 7.5% of the combined value of capitation and DTPs. The use of these indicators allows for comparability across institutions and private practices. The selected KPIs comprise drug prescribing, sick-leave rates, referrals to secondary care, referrals to primary care laboratories, and the proportion of preventive procedures performed, with the overall aim of reinforcing gatekeeping and promoting rational resource use. The Croatian Health Insurance Fund also defined two primary QIs: maintaining patient panels for chronic conditions (hypertension, diabetes, and chronic obstructive pulmonary disease [COPD]) and participation in peer-group counseling.

In addition, the Croatian Health Insurance Fund introduced the option of establishing informal “group practices,” further incentivized with a 5% bonus on the combined value of capitation and delivered services. The goals of the payment reform were to encourage family physicians to expand service provision, deliver care more efficiently and at higher quality, retain patients at the primary care level closer to their homes, and reduce referrals to hospitals. Additional objectives included increasing preventive check-ups, strengthening the management of chronic diseases, reducing antimicrobial resistance, and fostering improved communication and knowledge exchange among peers.

### Data analysis plan

We examined changes in ten outcome variables (the number of prescriptions issued by the family practitioner; the number of prescribed antibiotics; the number of referrals to secondary and tertiary health care; the number of referrals to laboratories; the number of patients with anticoagulant therapy determined within each period; the number of preventive check-ups performed; the number of patients with diabetes; the number of patients with hypertension; the number of patients with COPD; the number of patients undergoing ECG monitoring; the number of otoscopies; the number of abdomen ultrasounds; the number of rhinoscopies) before and after the payment change. All analyzed outcome variables are related to the new payment model and included in the incentivization scheme as explained above.

### Statistical analysis

Descriptive statistics (mean and standard deviation) are presented for each of the two family practitioners. For binary variables (numbers of newly reported cases) before and after payment change, frequencies are shown. Linear regression was conducted to investigate how demographic and health-related variables (sex, age, diagnosis, practice number) and their interactions with the period of measurement (before, after; excluding diagnosis, which remained the same in both periods) were related to the number of prescriptions and other outcome variables (number of prescribed antibiotics, number of referrals, etc). The analysis was performed in SPSS (IBM Corp., Armonk, NY, USA).

## Results

In order to assess the impact of the reform on the variables, we first analyzed the patient composition of the two family practices that participated in the study. Patients (189/3036) who did not visit the group practice from April 1, 2012 to March 31, 2014 were excluded. Out of 1261 patients who visited the first practitioner during the analyzed period, 53.6% were female, distributed in 6 age groups. There were 105 patients with diabetes, 500 with hypertension, and 102 with COPD. Out of 1586 patients who visited the second family practitioner during the analyzed period, 52.5% were female. There were 111 patients with diabetes, 526 with hypertension, and 119 with COPD (Table 1). The sex distribution of patients was similar across both practices, although the second practice had a larger overall patient population, particularly in the youngest age group (0-7 years).

**Table 1:** Sex and age of patients in the two studied practices

	Family practitioner practice number 1		Family practitioner practice number 2		Total	
	n	%	n	%	n	%
Sex						
male	585	46.4	754	47.5	1339	47.0
female	676	53.6	832	52.5	1508	53.0
Total	1261	100.0	1586	100.0	2847	100.0
Age						
0-7	46	3.6	140	8.8	186	6.5
8-18	129	10.2	148	9.3	277	9.7
19-45	396	31.4	518	32.7	914	32.1
46-64	381	30.2	450	28.4	831	29.2
65+	309	24.5	330	20.8	639	22.4
Total	1261	100.0	1586	100.0	2847	100.0

The second period was characterized by an increased number of prescriptions, laboratory referrals, preventive check-ups, electrocardiogram (ECG) monitoring, and otoscopy, alongside a decrease in hospital referrals and antibiotic prescriptions in both practices. The use of ultrasound diagnostics remained unchanged across practices (Table 2). The number of newly diagnosed cases of diabetes and chronic COPD increased, whereas new diagnoses of NCDs rose only in Practice 1. Practitioner 2 recorded fewer referrals overall; however, when mean values were compared, both practitioners demonstrated similar referral rates after the payment reform.

**Table 2:** Variables of family practitioners’ behavior in the period before and after payment change in two family practitioner practices

	Family practitioner practice number 1 (N = 1261)				Family practitioner practice number 2 (N = 1586)			
	before		after		before		after	
	mean	SD	mean	SD	mean	SD	mean	SD
Number of prescriptions	16.65	24.59	17.13	25.11	10.71	16.95	11.72	18.58
Number of prescribed antibiotics	0.49	1.09	0.45	0.99	0.49	1.00	0.47	0.99
Number of referrals	1.43	2.44	0.59	1.11	1.11	2.04	0.56	1.16
Number of referrals to laboratories	0.78	1.97	0.86	2.43	0.75	2.50	0.92	3.61
Dates of determining anticoagulant therapy	0.00	0.00	0.00	0.05	0.00	0.00	0.05	0.51
Number of preventive checkups	0.00	0.00	0.27	0.47	0.00	0.00	0.18	0.40
Provided service								
ECG monitoring	0.07	0.30	0.11	0.37	0.07	0.30	0.10	0.35
otoscopy	0.04	0.26	0.17	0.56	0.03	0.19	0.14	0.51
abdomen ultrasound	0.02	0.13	0.03	0.19	0.06	0.27	0.05	0.23
rhinoscopy	0.05	0.23	0.04	0.21	0.16	0.47	0.10	0.33
Diabetes discovered, n	6		9		7		6	
Hypertension discovered, n	42		38		41		31	
Chronic obstructive pulmonary disease discovered, n	4		11		8		6	

To further assess these patterns, a series of linear regression analyses were conducted to examine the predictive value of descriptive variables (age, sex, practice, and the presence of NCDs), as well as the interaction of these variables with age before and after the reform. A higher number of medications was prescribed for patients with diabetes, hypertension, and COPD. Significant interactions were observed between the post-intervention period and the age groups 0-7 and 45-64 years, showing a decrease in hospital referrals. They were also observed between the post-intervention period and the 65+ age group, indicating an increase in anticoagulant therapy and ECG services. Preventive examinations significantly increased across all age groups after the intervention (Supplemental Table 1 (Supplementary Table 1)and Supplemental Table 2(Supplementary Table 2)).

## Discussion

Following the 2013 payment reform in Croatia, hospital referrals decreased, while the provision of ECG monitoring, otoscopy, and abdominal ultrasounds increased. Also, the number of preventive check-ups significantly rose, accompanied by an overall increase in medication prescribing.

### Decrease in referring

One of the key components of the payment reform was the financial incentive to reduce hospital referrals, with the goals of avoiding unnecessary hospital visits, preventing duplication of procedures, reinforcing the role of family practitioners in managing referrals, and improving health care accessibility. This approach allowed patients to receive care for a broader range of conditions from their chosen family physicians, closer to home, and without long waiting times. In the studied group practice, the number of hospital referrals decreased following the reform, particularly for the more experienced, senior family practitioner. Evidence from managed health care systems suggests that introducing fundholding in primary care is associated with reduced hospital referrals (13). However, concerns have been raised that using financial incentives to limit service utilization may place physicians in ethical dilemmas, potentially resulting in the withholding of necessary care (14) and eroding trust between doctors and patients (15).

### Increase in providing some health services

Within the financing reform, unlike the previous payment scheme, a substantial portion of family physicians’ income was tied to fee-for-service, which may explain the observed increase in the provision of ECG monitoring, otoscopy, and abdominal ultrasound services following the reform. Multiple studies indicate that combining fee-for-service with performance-based payments can lead primary care physicians to deliver a higher volume of services, manage more patients, reduce referrals, and care for more complex cases, particularly compared with traditional fee-for-service models alone (16-18).

One study found that implementing financial incentives improved documentation of diabetes care quality, although it did not translate into better patient management or health outcomes (19). Another study reported that introducing a contract with financial incentives for general practitioners led to a significant increase in the documentation of quality parameters for stroke patients (20). In the UK, the implementation of pay-for-performance and quality incentive programs in primary care was associated with improved and more equitable management of coronary heart disease across different ethnic groups (21), a substantial increase in documentation of chronic heart disease-related quality indicators (22), and enhanced monitoring of diabetic patients and related quality metrics (23). However, one study noted that while the UK payment reform between 2001 and 2007 significantly improved incentivized quality indicators, there were minor negative effects on non-incentivized areas of care (24). Overall, research on the impact of different payment models on family physicians’ service provision remains limited (25).

### Increase in providing prevention

The analysis revealed a significant improvement in the provision of preventive check-ups in the Breznica group practice, which may be attributed not only to the financial incentives introduced by the reform but also to the implementation of IT tools (panels) that facilitate recording during preventive visits. One study examined how four different payment models — fee-for-service, capitation, general practitioner fundholding, and a penalty-based approach — affect general practitioners’ decisions regarding preventive care, framing it as a strategic choice rather than a direct response to financial incentives. In these models, preventive services are delivered based on a balance between potential cost savings from reduced unnecessary referrals and the potential downside of increased patient waiting times (26,27). Another study reported a substantial increase in the number of patients receiving recorded smoking cessation advice following the introduction of the UK payment reform (28). Conversely, a different study found that pay-for-performance programs had only a modest positive effect on preventive measures, such as immunization rates (29).

### Increase in prescribing medicines

Drug prescribing increased in the studied group practice, despite financial incentives designed to reduce prescription numbers. The practice team attributed this trend to a higher patient volume, with 16 635 visits recorded in the second period compared with 15 623 in the first period, representing a 6% increase. This rise may reflect the additional effort of family physicians, also incentivized to manage patients at the primary care level. The increase in prescriptions was particularly pronounced among patients with chronic diseases, likely facilitated by the implementation of panels for chronic disease management and the associated financial incentives. Supporting evidence from the UK indicates that medications included in the incentive scheme experienced a more rapid rise in prescription rates than non-incentivized drugs, both before and after the introduction of the new contract (30).

Given the limited evidence regarding the impact of financial incentives on reducing referrals – available primarily from studies on fundholding in primary care – and the scarcity of research on increased prescribing despite incentives to reduce it, these findings should be considered preliminary. Further investigation using more rigorous study designs is required to confirm these effects.

### Limitations and conclusions

Several study limitations must be mentioned. First, this exploratory case study covers only one rural practice actively involved in designing the reform, which introduces selection bias. Second, the sample size (3036 patients; two family physicians) is very small compared with the national context. Third, baseline reporting of preventive services was poor, limiting the reliability of observed improvements. Fourth, no control group was included, and the one-year observation period is insufficient to capture sustainable trends or seasonal effects. Fifth, the data are 10 years old; however, the research intentionally analyzed one year before and after the reform, and no significant changes in the payment scheme or recent studies on the issue have been done by now.

In conclusion, the payment reform appears to have positively affected the performance of family physicians in a front-runner rural group practice in Croatia. The number of services provided to patients increased, particularly preventive check-ups, while the number of referrals to hospitals decreased, which suggests an improvement in the quality of care that aligns with the reform’s objectives. Furthermore, evaluating improvements in medical outcomes in relation to financial incentives could provide an even stronger indicator of enhanced quality of care; however, this aspect was not included in Croatia’s financial reform. The observed increase in prescribing, despite existing disincentives, may reflect physicians’ efforts to manage patients at the primary care level, resulting in more visits. This, however, underscores the complexity of the relationship between incentives and physician behavior in primary health care. These findings, derived from the analysis of a single rural group practice, should be considered preliminary and primarily intended to generate hypotheses and guide future, more rigorous longitudinal and multi-site studies.
