# Supplementary material for: The impact of Croatia’s 2013 Primary Care Payment Reform: an exploratory case study of a rural family practitioner group practice
Source: Croat Med J. 2025 Oct;66(5):360–6. doi: 10.3325/cmj.2025.66.360 (PMC12623254; doi:10.3325/cmj.2025.66.360)
Supplement: Supplementary Table 1 [file CroatMedJ_66_s006.pdf]

Supplemental Table 1. Results of linear regression for criteria variables of family practitioners' behavior

Predictor	Number of prescriptions			Number of prescribed antibiotics			Number of referrals			Number of referrals to labs			Dates of determining anticoagulant therapy		
	B	*P*	SE	B	*p*	SE	B	*p*	SE	B	*p*	SE	B	*p*	SE
Constant	**3.873**	**<0.001**	0.740	**0.306**	**<0.001**	0.044	**1.077**	**<0.001**	0.077	**0.322**	**0.007**	0.120	-0.001	0.946	0.012
Sex (0=male, 1=female)	**2.040**	**0.001**	0.639	**0.178**	**<0.001**	0.038	-0.070	0.290	0.066	0.072	0.489	0.104	0.000	0.969	0.010
Period (0=before, 1=after)	0.092	0.930	1.042	0.005	0.930	0.062	**-0.696**	**<0.001**	0.108	0.007	0.967	0.169	-0.032	0.055	0.017
Age 0-7 (0=no, 1=yes)	1.366	0.314	1.356	**0.365**	**<0.001**	0.081	-0.182	0.194	0.140	0.198	0.369	0.220	0.000	0.993	0.022
Age 8-18 (0=no, 1=yes)	-0.889	0.440	1.151	**0.161**	**0.019**	0.069	0.019	0.872	0.119	0.001	0.998	0.187	-0.001	0.950	0.019
Age 19-45 (0=no, 1=yes)															
Age 46-64 (0=no, 1=yes)	**4.274**	**<0.001**	0.846	-0.033	0.508	0.051	**0.663**	**<0.001**	0.088	**0.312**	**0.023**	0.137	0.003	0.846	0.014
Age 65 and more (0=no, 1=yes)	**12.205**	**<0.001**	0.980	0.001	0.993	0.058	0.161	0.112	0.101	**0.409**	**0.010**	0.159	0.002	0.886	0.016
Patient has diabetes (0=no, 1=yes)	**17.673**	**<0.001**	0.882	0.062	0.236	0.053	**0.656**	**<0.001**	0.091	0.249	0.082	0.143	-0.012	0.414	0.014
Patient has hypertension (0=no, 1=yes)	**13.206**	**<0.001**	0.619	**0.079**	**0.032**	0.037	**0.189**	**0.003**	0.064	**0.450**	**<0.001**	0.100	-0.005	0.634	0.010
Patient has COPD (0=no, 1=yes)	**9.044**	**<0.001**	0.845	**0.422**	**<0.001**	0.050	**0.321**	**<0.001**	0.087	0.188	0.171	0.137	**0.027**	**0.046**	0.014

Practice (0=practice number 1, 1=practice number 2)	**-4.320**	**<0.001**	0.637	-0.010	0.790	0.038	**-0.267**	**<0.001**	0.066	0.015	0.888	0.103	0.000	0.986	0.010
Period × sex	-0.202	0.823	0.902	-0.062	0.246	0.054	-0.013	0.887	0.093	-0.030	0.836	0.146	0.005	0.705	0.015
Period × age 0-7 y	0.103	0.957	1.917	0.009	0.939	0.114	**0.522**	**0.008**	0.198	0.054	0.861	0.311	-0.011	0.730	0.031
Period × age 8-18 y	0.323	0.843	1.627	0.097	0.316	0.097	0.174	0.300	0.168	0.237	0.369	0.264	0.001	0.981	0.026
Period × age 19-45 y*															
Period × age 46-64 y	1.631	0.151	1.137	-0.071	0.294	0.068	**-0.417**	**<0.001**	0.118	0.073	0.694	0.184	0.035	0.053	0.018
Period × age 65+ y	-0.125	0.919	1.233	-0.008	0.910	0.074	-0.189	0.139	0.127	0.181	0.365	0.200	**0.087**	**<0.001**	0.020
Period × practice	0.553	0.539	0.900	0.023	0.670	0.054	0.246	0.080	0.093	0.100	0.495	0.146	**0.054**	**<0.001**	0.014
F		224.087; *p* = <0.001			11.303; *p* = <0.001			30.006; *p* = <0.001			80.293; *p* = <0.001			50.898; *p* = <0.001	
R^2		0.387			0.031			0.078			0.023			0.016	
Adjusted R^2		0.385			0.028			0.075			0.020			0.014	

*reference category.
