# Supplementary material for: The impact of Croatia’s 2013 Primary Care Payment Reform: an exploratory case study of a rural family practitioner group practice
Source: Croat Med J. 2025 Oct;66(5):360–6. doi: 10.3325/cmj.2025.66.360 (PMC12623254; doi:10.3325/cmj.2025.66.360)
Supplement: Supplementary Table 2 [file CroatMedJ_66_s007.pdf]

Supplemental Table 2. Results of linear regression for criteria variables of family practitioners' behavior additional categories

Predictor	Number of preventive checkups			Provided service ECG monitoring			Provided service otoscopy			Provided service abdomen ultrasound			Provided service rhinoscopy		
	B	*p*	SE	B	*P*	SE	B	*p*	SE	B	*p*	SE	B	*p*	SE
Constant	0.009	0.501	0.013	0.026	0.065	0.014	0.031	0.076	0.018	-0.015	0.126	0.009	**0.060**	**<0.001**	0.015
Sex (0=male, 1=female)	0.001	0.899	0.011	**0.028**	**0.022**	0.012	-0.012	0.413	0.015	**0.028**	**<0.001**	0.008	0.021	0.093	0.013
Period (0=before, 1=after)	**0.328**	**<0.001**	0.018	0.022	0.270	0.020	**0.079**	**0.001**	0.025	0.025	0.058	0.013	-0.008	0.713	0.021
Age 1-7 (0=no, 1=yes)	-0.007	0.764	0.024	-0.048	0.070	0.026	-0.020	0.535	0.032	-0.033	0.058	0.017	**-0.131**	**<0.001**	0.027
Age 8-18 (0=no, 1=yes)	-0.006	0.781	0.020	**-0.048**	**0.034**	0.022	-0.015	0.593	0.027	-0.015	0.314	0.015	0.032	0.161	0.023
Age 19-45 (0=no, 1=yes)															
Age 46-64 (0=no, 1=yes)	**0.036**	**0.016**	0.015	0.008	0.643	0.016	-0.001	0.968	0.020	**0.039**	**<0.001**	0.011	**-0.036**	**0.030**	0.017
Age 65 and more (0=no, 1=yes)	**0.063**	**<0.001**	0.017	-0.017	0.362	0.019	0.022	0.347	0.023	0.023	0.068	0.013	**-0.109**	**<0.001**	0.019

Patient has diabetes (0=no, 1=yes)	-0.026	0.093	0.016	-0.021	0.226	0.017	**0.058**	**0.005**	0.021	-0.011	0.325	0.011	**-0.043**	**0.014**	0.017
Patient has hypertension (0=no, 1=yes)	**-0.078**	**<0.001**	0.011	**0.082**	**<0.001**	0.012	0.013	0.366	0.015	0.009	0.271	0.008	**0.030**	**0.014**	0.012
Patient has COPD (0=no, 1=yes)	-0.024	0.108	0.015	**0.044**	**0.007**	0.016	0.016	0.417	0.020	0.009	0.403	0.011	**0.056**	**<0.001**	0.017
Practice (0=practice number 1, 1=practice number 2)	-0.002	0.844	0.011	0.011	0.372	0.012	-0.008	0.575	0.015	**0.047**	**<0.001**	0.008	**0.124**	**<0.001**	0.013
Period × gender	-0.017	0.271	0.016	-0.004	0.828	0.017	0.024	0.271	0.021	-0.002	0.863	0.012	0.001	0.938	0.018
Period × age 0-7 y	**0.114**	**<0.001**	0.034	-0.007	0.856	0.037	**0.512**	**<0.001**	0.045	-0.008	0.740	0.025	0.044	0.249	0.038
Period × age 8-18 y	**0.174**	**<0.001**	0.029	0.004	0.898	0.031	**0.209**	**<0.001**	0.039	-0.016	0.434	0.021	-0.010	0.758	0.032
Period × age 19-45 y*															
Period × age 46-64 y	**-0.075**	**<0.001**	0.020	0.021	0.343	0.022	-0.006	0.836	0.027	-0.013	0.386	0.015	-0.011	0.637	0.023
Period × age 65+ y	**-0.194**	**<0.001**	0.022	**0.082**	**<0.001**	0.024	0.005	0.878	0.029	-0.024	0.133	0.016	0.010	0.696	0.024

Period × practice	**-0.106**	**<0.001**	0.016	-0.018	0.290	0.017	-0.040	0.059	0.021	-0.022	0.060	0.012	**-0.063**	**<0.001**	0.018
F		820.872; *p* = <0.001			140.092; *p* = <0.001			270.279; *p* = <0.001			70.886; *p* = <0.001			150.584; *p* = <0.001	
R^2		0.189			0.038			0.072			0.022			0.042	
Adjusted R^2		0.187			0.035			0.069			0.019			0.039	

*reference category.
